# Preoperative localization of pulmonary nodules near the fissures: electromagnetic navigation bronchoscopy vs. hook-wire percutaneous localization

**DOI:** 10.3389/fsurg.2026.1803258

**Published:** 2026-03-25

**Authors:** Yuhui Gong, Shiyu Shen, Jialiang Liu, Haitao Huang

**Affiliations:** 1 Department of Cardiothoracic Surgery, The Fourth Affiliated Hospital of Soochow University, Suzhou, Jiangsu, China; 2 Department of Thoracic Surgery, The First Affiliated Hospital of Soochow University, Suzhou, Jiangsu, China

**Keywords:** electromagnetic navigation bronchoscopy, hook-wire, ICG, preoperative location, pulmonary nodules near the fissures

## Abstract

**Background:**

Accurate preoperative localization is crucial to improving the success rates of thoracoscopic surgery and minimizing postoperative complications. Hook-wire localization can be inaccurate when marking small pulmonary nodules near the fissures of the lungs. This study aims to evaluate the accuracy and safety of indocyanine green (ICG) staining combined with electromagnetic navigation bronchoscopy (ENB) compared to hook-wire percutaneous localization for pulmonary nodules near the fissures.

**Methods:**

A retrospective analysis was conducted on 64 patients who underwent video-assisted thoracic surgery (VATS). The patients were divided into two groups: Group A (ENB with ICG staining) and Group B (hook-wire percutaneous localization). The two groups were compared for localization time, complication rates, specimen retrieval time, and the need for extended resection.

**Results:**

The ENB group required more time for localization than the hook-wire group (25.30 min vs. 19.68 min, *P* < 0.05). However, only one case in the ENB group required further resection (3.0% vs. 22.6%, *P* = 0.018). The incidence of pneumothorax was significantly lower in the ENB group than in the hook-wire group (6.10% vs. 25.80%, *P* = 0.03).

**Conclusion:**

ENB combined with ICG staining for localization of nodules near the fissures significantly reduces the likelihood of extended resection and is associated with a higher safety profile compared to hook-wire percutaneous localization.

## Introduction

Lung cancer remains the leading cause of cancer-related mortality worldwide, posing a significant threat to human health (1). With the increasing use of low-dose spiral CT (LDCT) scans, more peripheral solitary pulmonary nodules (PSPNs) and ground-glass nodules (GGNs) are being detected. These pulmonary nodules may have malignant potential and require surgical resection (2). Surgical excision and pathological examination are the standard modes of diagnosis and treatment for pulmonary nodules. However, thoracoscopic surgery is often limited by visual, tactile, and operational space constraints, particularly when identifying small nodules, especially those with low density or located deep within the lung. Preoperative localization of pulmonary nodules, particularly for those located near fissures, remains a significant challenge for thoracic surgeons.

Several methods are available for localization, including CT-guided hook-wire percutaneous localization, which is widely used due to its simplicity, high accuracy, and short learning curve (3). In contrast, percutaneous placement of spring coils, similar to hook-wire, may result in deep placement, making it difficult to access during surgery. This often requires the use of a C-arm, increasing radiation exposure and complicating the surgical process. Recent technical advances have shown the feasibility of percutaneous and hybrid operating room-based marking techniques using indocyanine green (ICG), which may be combined with direct lesion marking (4). Electromagnetic navigation bronchoscopy (ENB) (5), combined with bronchoscopic techniques, electronic navigation, and 3D reconstruction, allows for deep access (up to the 14th generation of the distal bronchi) and provides accurate localization of pulmonary nodules. Emerging robotic navigation technology offers the advantage of continuous visualization and stability for detailed operations (6).

**Figure 1:**
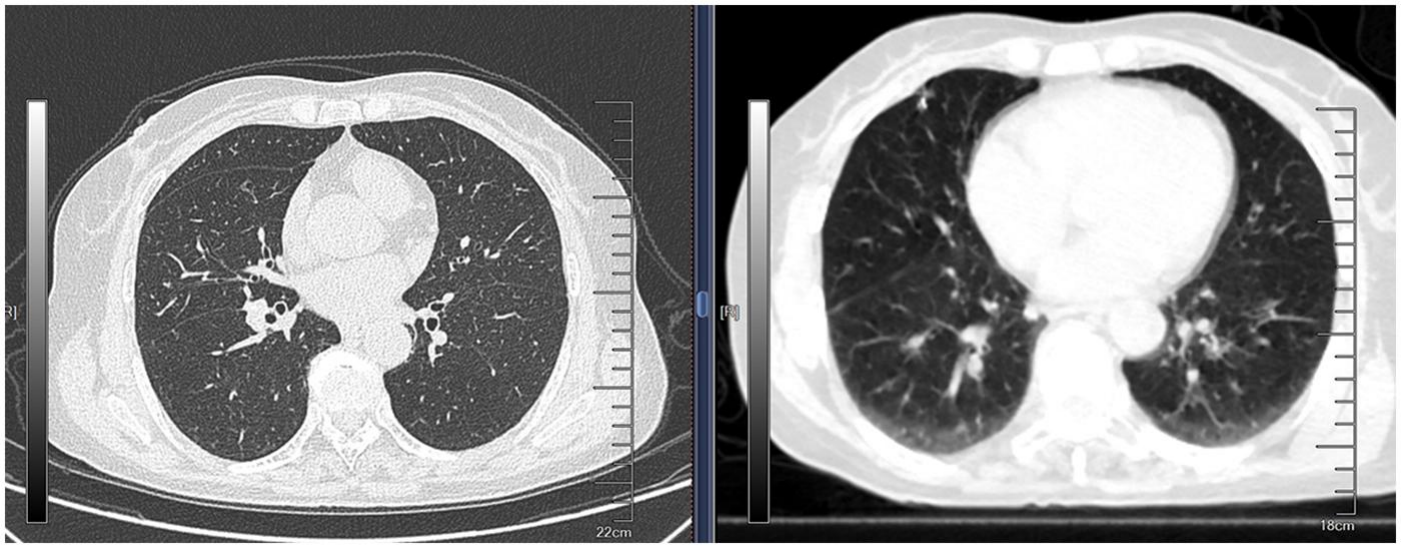
Hook-wire localization of a right middle lobe nodule: the nodule is remote from the chest wall and obscured anteriorly by the interlobar fissure, with the hook-wire tip positioned inferior to the nodule.

**Figure 2:**
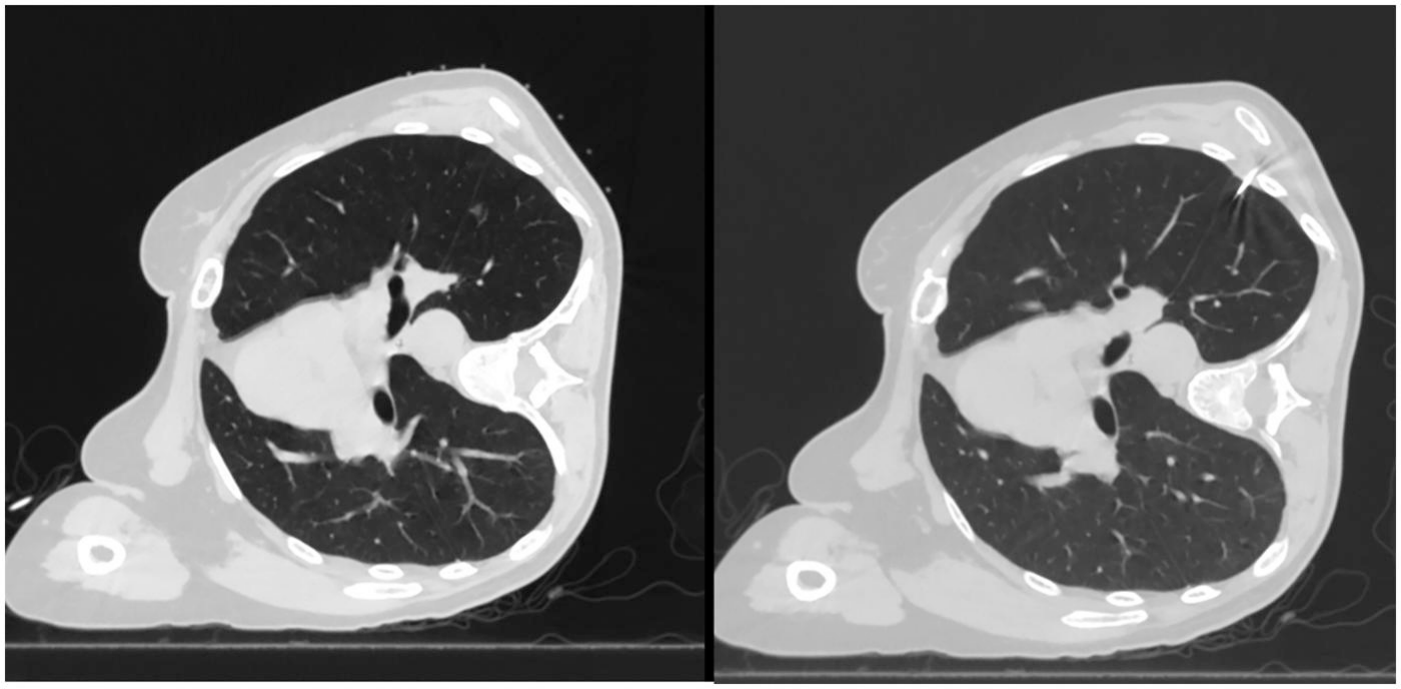
Hook-wire localization of a left lower lobe nodule: the nodule is remote from the chest wall, with a significant distance from the puncture site.

**Figure 3:**
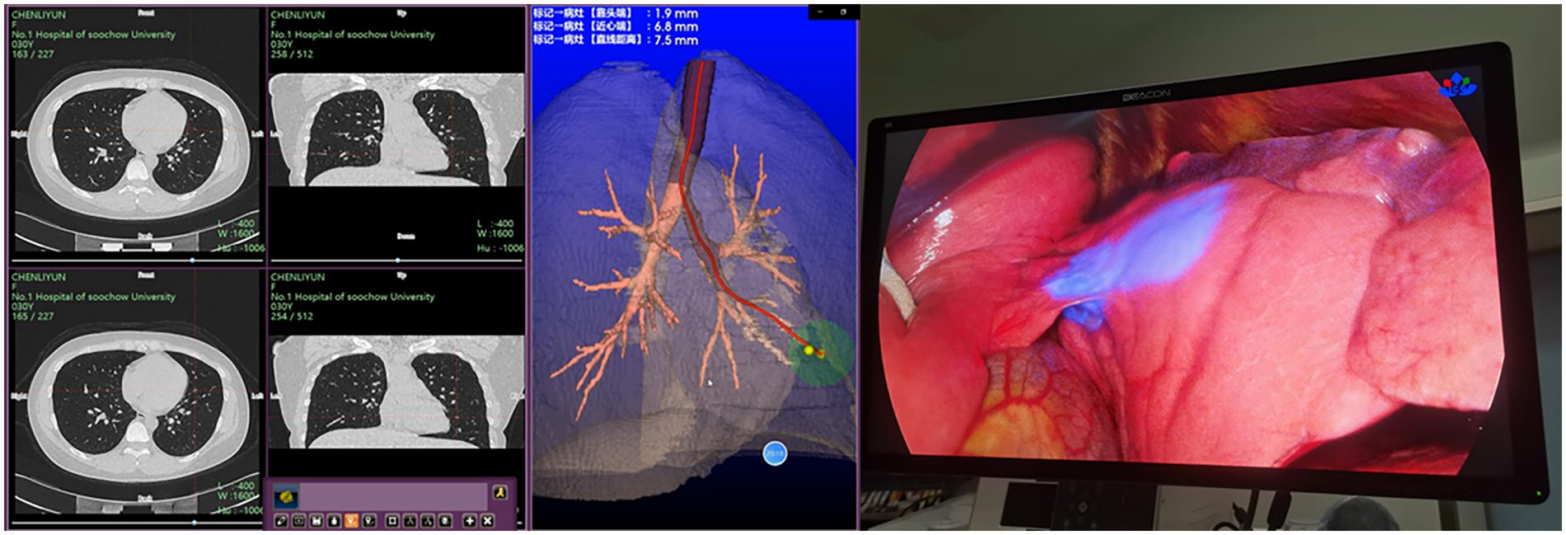
ENB localization of a left lower lobe nodule: a well-defined ICG staining spot identified at the interlobar fissure.

Nodules located near the fissures of the lungs are often adjacent to the visceral pleura and can theoretically be removed through wedge resection (7, 8). However, because these nodules are far from the chest wall, CT-guided hook-wire localization only positions the needle as close as possible to the lung tissue near the chest wall, yielding a relative position and distance that is prone to inaccuracy (Figures 1, 2). Due to the movement of the lungs during surgery and the uneven collapse of different areas of the lungs, it is difficult to ensure the accuracy and reliability of this relative position. Based on such vague positioning points, on the one hand, it may lead to an inability to guarantee a safe margin (the distance between the tumor and the margin is >2 cm), and on the other hand, it may lead to the forced expansion of the resection range (segmentectomy, or even lobectomy). In a worse case, during the resection, the cutting stapler cuts the tumor itself, which may cause the possibility of tumor implantation and metastasis. ENB, using a guidewire within the bronchial tree, allows for accurate positioning close to the pulmonary nodule (Figure 3). Studies comparing these two methods for preoperative localization of nodules near the fissures are limited. This study aims to investigate further.

## Methods

### Inclusion and exclusion criteria

This retrospective cohort study included 64 patients who underwent thoracoscopic surgery between October 2023 and May 2025 at the Fourth and First Affiliated Hospitals of Soochow University. The study was approved by the institutional review board (IRB) of the Fourth Affiliated Hospital of Soochow University (IRB number: 251182). Written informed consent was obtained from each patient or their legal guardian.

### Inclusion criteria

1. Age ≥ 18 years.
2. Patients with suspected primary lung cancer based on clinical and imaging features
3. Preoperative diagnosis of a pulmonary nodule located within 5 mm (≤ 0.5 cm) from the adjacent fissure and at least 20 mm from the chest wall, with indications for wedge resection via thoracoscopy.
4. Nodule diameter ≤ 20 mm, located in the outer third of the lung, difficult to locate visually or palpably.
5. No contraindications for pulmonary surgery or anesthesia.

### Exclusion criteria

1. Age < 18 years.
2. Patients with a history of extrapulmonary malignancy or clinically suspected metastatic pulmonary nodules
3. Nodules not located near the fissures or close to the chest wall (<20 mm); unsuitable for wedge resection or requiring lobectomy.
4. Nodule diameter >20 mm, located in the central two-thirds of the lung, and easy to locate visually or palpably.
5. Presence of surgical or anesthetic contraindications.
6. Preoperative distant metastasis or uncontrolled infectious diseases.

## Electromagnetic navigation bronchoscopy with ICG staining

### Equipment and principles

The electromagnetic navigation bronchoscopy (ENB) system [model ZhuHai Shi VB-3342 (4.2–2.0)] and its associated accessories were provided by LungCare (China). The core system comprises the following four main components:

1. Medical Imaging Workstation: A computer with navigation software that imports high-resolution CT data in DICOM format, creating a 3D image and planning the navigation path.
2. Electromagnetic Flat Plate Generator: A device that emits electromagnetic signals, placed under the patient's back, creating a magnetic field for real-time localization data.
3. Locatable Wire (LW): A thin guidewire (1.15 mm) with an electromagnetic sensor at the tip, enabling precise positioning within the bronchial tree.
4. Guiding Sheath (GS): A sheath used to guide the locatable wire into the bronchial tree for accurate navigation.

### Procedure

1. Registration and Matching: High-resolution CT images are imported into the navigation system, which generates a virtual bronchial tree. The patient is placed in the supine position, intubated, and under general anesthesia. The electromagnetic plate is placed under the patient's back, and sensors are attached to the chest. Using a thin bronchoscope, the operator manipulated the guide sheath and locatable guide through the main bronchi, left/right bronchi, and segmental bronchi to complete system registration, matching it with the pre-procedurally simulated 3D bronchial reconstruction.
2. Navigation: The bronchoscope is advanced to the distal bronchus, with the guiding sheath and locatable wire gradually progressing to the nodule's location. ICG is injected for localization, and the fluorescence is observed under the thoracoscope.
3. ICG Staining: After localization, ICG (0.25 mg/mL, 2.2–2.3 mL) is injected into the guiding sheath to mark the nodule.

### Hook-Wire percutaneous localization

The patient undergoes a CT scan, and the hook-wire (GALLINI S.R.L, Model: BLR18/10, 18G*10 cm) is inserted into the lung using a preplanned insertion site. CT scans confirm the position of the hook-wire near the nodule, with double hooks released to secure the nodule's location. The patient is closely monitored for pneumothorax and other complications. If necessary, chest tube drainage is performed.

### Surgical methods

Both groups underwent thoracoscopic surgery. In the ENB group, the surgeon used the fluoroscopy mode to identify the stained nodule. In the hook-wire group, the surgeon used white light thoracoscopy to identify the nodule based on the position of the puncture needle. If the nodule could not be identified intraoperatively or was absent from the resected specimen, an anatomical segmentectomy or wider resection was performed based on preoperative anatomical mapping and surgical judgment.

### Observation parameters

1. Localization Time: Time from wire-magnetic field matching to ICG injection (ENB group) and from the first to last CT scan (hook-wire group).
2. Need for Extended Resection: Extended resection (defined as additional margin resection, segmentectomy, or lobectomy beyond the initial planned wedge) was performed if: a) post-resection pathological examination of the specimen revealed an insufficient microscopic safety margin (<2 cm) or no identifiable nodule; or b) the surgeon intraoperatively deemed the localization marker (dye or wire) unreliable and proceeded directly with a larger anatomic resection.
3. Pneumothorax Occurrence: In the ENB group, pneumothorax was defined as significant lung collapse observed upon thoracic entry during VATS with concomitant visual confirmation of pleural disruption attributed to the navigation procedure. In the hook-wire group, pneumothorax was radiologically defined by its presence on the final CT scan, identified as lung collapse accompanied by an air-filled space devoid of lung markings.

### Statistical analysis

Data were analyzed using SPSS software. Continuous variables were compared using t-tests or rank-sum tests, and categorical variables were analyzed with chi-square tests.

## Results

### Patient characteristics

A total of 64 patients were included, with 33 in the ENB group and 31 in the hook-wire group. There were no significant differences between the groups in terms of age, sex, nodule size, or distance from the chest wall (all ***P*** > 0.05), confirming baseline comparability (Table 1).

**Table 1:** Patient characteristics and detailed nodule parameters between two groups.

Variables	ENB (*n* = 33)	Hook-wire (*n* = 31)	Statistic	***P***
Gender, *n* (%)			χ^2^ = 0.071	0.790
Male	16（48.50）	14（45.20）		
Female	17（51.50）	17（54.80）		
Age (y), Mean ± SD	47.09 ± 9.869	46.94 ± 8.656	t = 0.607	0.947
Height (cm), Mean ± SD	165.94 ± 5.690	166.74 ± 5.079	t = −0.594	0.555
Weight (kg), Mean ± SD	62.91 ± 5.603	63.10 ± 3.944	t = −0.156	0.877
Maximum nodule diameter (mm) M (Q₁, Q₃)	8.00（7.00,10.00）	7.00（7.00,10.00）	Z = −0.553	0.580
Depth of nodule from chest wall (mm), M (Q₁, Q₃)	26.00（24.50,30.00）	27.00 (25.00,30.00)	Z = −0.353	0.724
Nodule location, *n* (%)			χ^2^ = 0.707	0.950
Right upper lobe	8	8		
Right middle lobe	4	4		
Right lower lobe	6	7		
Left upper lobe	8	5		
Left lower lobe	7	7		
Radiological pattern, *n* (%)			χ^2^ = 0.019	0.991
Pure ground glass nodule	23	22		
Part solid nodule	9	8		
Solid nodule	1	1		

### Outcome comparison

The results of the two groups’ comparison are shown in Table 2. The mean localization time was significantly longer in the ENB group compared to the hook-wire group (25.30 ± 4.2 min vs. 19.68 ± 3.8 min, ***P*** < 0.05). However, the ENB group demonstrated a markedly lower rate of extended resection (3.0% vs. 22.6%, ***P*** = 0.018), indicating more accurate localization and more frequent successful wedge resections. Furthermore, the incidence of pneumothorax was significantly lower in the ENB group (6.10% vs. 25.80%, ***P*** = 0.03). Specimen retrieval time was comparable between the two groups.

**Table 2:** Comparison of efficacy and safety outcomes between the two groups.

Variables	ENB (*n* = 33)	Hook-wire (*n* = 31)	Statistic	***P***
Localization duration (min) Mean ± SD	25.30 ± 2.834	19.68 ± 3.31	t = 7.317	<0.05
Specimen retrieval time (min) Mean ± SD	20.30 ± 3.157	19.32 ± 2.761	t = 1.391	0.192
Pneumothorax rate, *n* (%)	2 (6.10)	8 (25.80)	χ^2^ = 4.727	0.03
Extended resection, *n* (%)	1 (3.00)	7 (22.60)	χ^2^ = 5.586	0.018

## Discussion

The selection of a preoperative localization method for pulmonary nodules should be based on a comprehensive evaluation of safety, accuracy, and cost. The traditional Hook-wire technique remains a definitive and effective method and is the most widely used in clinical practice. AR-assisted localization (9) allows surgeons to directly “see” the nodule's position intraoperatively, thereby avoiding or reducing the need for preoperative percutaneous puncture procedures. Robotic-assisted localization systems reduce the number of “trial-and-error” attempts associated with traditional manual puncture and significantly lower radiation exposure (10). Robotic bronchoscopy (11) offers a new, non-invasive option for localization via a natural transluminal approach. For patients at high risk for traditional percutaneous puncture (e.g., nodules obscured by the scapula or adjacent to major vessels), robotic bronchoscopy can reach more peripheral bronchi to perform precise dye marking. The above-mentioned three localization devices are relatively expensive and are not yet widely used in clinical practice. Hybrid OR-based localization (4) using a dual marking technique with Lipiodol and indocyanine green (ICG) under cone-beam CT guidance represents a significant and relevant advancement. This approach offers distinct advantages, including real-time imaging, the ability to perform localization and resection in a single anesthetic session, and the avoidance of patient transfer, thereby potentially reducing the risk of marker dislodgement and streamlining the surgical workflow.

For pulmonary nodules located near the fissures, their characteristics—being subpleural yet distant from the chest wall—make precise localization via percutaneous hook-wire placement challenging (12). This study retrospectively compared the safety and accuracy of ENB-guided marking vs. hook-wire localization for such challenging cases. The findings indicate that ENB-guided marking improves localization accuracy, reduces the probability of requiring extended resections, enhances procedural safety, and helps preserve more lung tissue. This advantage stems from the integration of electromagnetic navigation with 3D reconstruction technology, using an ultrathin flexible bronchoscope as a conduit. This approach visualizes, provides real-time feedback for, and achieves comprehensive access to the localization pathway (13, 14). In the study by Mariolo et al. (15), the median duration for ENB-guided localization was 25 min (range: 19–33 min), with a procedural success rate as high as 94%. Similarly, Fu et al. (16). compared ENB with hook-wire localization and found that the ENB approach demonstrated higher success rates, fewer complications, and a lower incidence of adverse events, supporting its role as a promising, safe, and feasible localization method for challenging pulmonary nodules. Their study benefited from a relatively large dataset for comparative analysis; however, it did not stratify or compare pulmonary nodules based on their specific locations, representing a limitation. Further, Yuan Xu et al. (17)evaluated the efficacy and safety of four ENB-guided ICG marking techniques: direct lesion marking, surface marking, resection margin marking, and perinodular sphere marking. Their results indicated that ENB-guided ICG marking combined with fluorescence-assisted thoracoscopic surgery is a safe and effective preoperative localization technique for deep-seated pulmonary nodules. In particular, surface marking and resection margin marking provided reliable and precise guidance for the surgical resection of such nodules.

Our comparative analysis also revealed that during ENB-guided dye marking, careful attention must be paid to the distance between the guidewire tip and the visceral pleura. Excessive insertion depth of both the guidewire and the guiding sheath may risk pleural penetration, leading to pneumothorax, or cause extravasation of ICG into the pleural cavity, contaminating the surgical field. One case in our series exemplifies this, where the localization site was identified based on a pleural breach, but resulted in intraoperative field contamination. Conversely, insufficient insertion depth may lead to incomplete or diffuse dye deposition, making the target difficult to identify during surgery. Ensuring procedural stability requires excellent coordination between the surgeon and the technical assistant. Furthermore, after the surgeon identifies the target position and suspends hand movement while holding the bronchoscope, an assistant must stabilize both the endotracheal tube and the bronchoscope to prevent displacement of the localization site. The implementation of a specialized fixation device at the bronchoscope port to secure its position could potentially further minimize displacement and improve localization accuracy.

Our investigation into localization methods for nodules near the fissures demonstrates that ENB-guided marking offers distinct advantages in terms of safety and accuracy. Additionally, ENB localization is performed under general anesthesia, eliminating procedure-related pain and obviating the need for post-localization transport and waiting time before surgery, thereby avoiding potential delayed complications associated with percutaneous techniques. In current clinical practice, hook-wire localization remains widely used due to its low cost, relative ease of operation, and high accuracy. However, for nodules in other specific challenging locations, ENB-guided localization may represent a superior alternative. Future research could involve a comprehensive analysis integrating factors such as segmental anatomy, peripheral bony structures, proximity to major vessels, and fissures. Combining this with artificial intelligence analysis may enable the creation of individualized “mapping zones” on the lung surface to guide the optimal choice of localization technique in subsequent clinical practice.

Our study has several limitations. First, this study is limited by its modest sample size (*n* = 64) and the low overall incidence of adverse events. For example, only one patient in the ENB group required extended resection, and pneumothorax rates were relatively low in both groups. These factors reduce the statistical power of our analyses, potentially masking true differences in uncommon outcomes. Larger, multi-center studies are needed to confirm our findings and to provide more precise estimates of treatment effects and complication rates. Second, A key limitation is the differing pneumothorax definitions between groups. In the ENB group, pneumothorax was assessed upon VATS entry, where iatrogenic pneumothorax is present, obscuring small procedure-related air leaks. Conversely, hook-wire patients underwent immediate post-procedural CT, which is highly sensitive for detecting trace pneumothorax. This discrepancy likely caused underdetection of minor pneumothoraces in the ENB group, introducing misclassification bias. Thus, the observed difference in pneumothorax rates should be interpreted cautiously. Future studies should adopt standardized post-procedural radiographic assessment for both groups. Furthermore, this study has several limitations inherent to its retrospective, non-randomized design. First, patients were not randomly assigned to the two localization techniques; allocation was based on the availability of the ENB system over different time periods. This sequential allocation introduces potential selection bias, as unmeasured differences in patient characteristics, surgical expertise, or perioperative management between the two time periods may have influenced the outcomes. Although baseline characteristics were comparable between groups, the possibility of residual confounding cannot be entirely excluded. Prospective randomized controlled trials are needed to confirm our findings and provide a higher level of evidence. Notably, the two groups differed fundamentally in patient status: ENB under general anesthesia vs. hook-wire in awake, spontaneously breathing patients. This affects pneumothorax rates: percutaneous puncture has a higher baseline risk, and pneumothorax in non-intubated patients is more readily detected and clinically meaningful, while under anesthesia it may be masked. Thus, the higher hook-wire pneumothorax rate is at least partly due to these inherent differences, not solely localization accuracy. Future studies should standardize assessment for fairer comparisons.

## Conclusion

ENB with ICG staining appears to be associated with lower extended resection rates and fewer clinically detected pneumothoraces compared with hook-wire percutaneous localization for nodules near the fissures. As ENB and related technologies become more widely available, their role in clinical practice is expected to expand.

## Data Availability

The original contributions presented in the study are included in the article/Supplementary Material, further inquiries can be directed to the corresponding authors.
